# Genetic overlap between Parkinson’s disease and inflammatory bowel disease

**DOI:** 10.1093/braincomms/fcad002

**Published:** 2023-01-03

**Authors:** Xiaoying Kang, Alexander Ploner, Yunzhang Wang, Jonas F Ludvigsson, Dylan M Williams, Nancy L Pedersen, Karin Wirdefeldt

**Affiliations:** Department of Medical Epidemiology and Biostatistics, Karolinska Institutet, SE-17165 Stockholm, Sweden; Department of Medical Epidemiology and Biostatistics, Karolinska Institutet, SE-17165 Stockholm, Sweden; Department of Medical Epidemiology and Biostatistics, Karolinska Institutet, SE-17165 Stockholm, Sweden; Department of Medical Epidemiology and Biostatistics, Karolinska Institutet, SE-17165 Stockholm, Sweden; Department of Pediatrics, Örebro University Hospital, SE-70116 Örebro, Sweden; Department of Medical Epidemiology and Biostatistics, Karolinska Institutet, SE-17165 Stockholm, Sweden; MRC Unit for Lifelong Health and Ageing at UCL, University College London, London WC1E 7HE, UK; Department of Medical Epidemiology and Biostatistics, Karolinska Institutet, SE-17165 Stockholm, Sweden; Department of Medical Epidemiology and Biostatistics, Karolinska Institutet, SE-17165 Stockholm, Sweden; Department of Clinical Neuroscience, Karolinska Institutet, SE-17177 Stockholm, Sweden

**Keywords:** Parkinson’s disease, inflammatory bowel disease, genetic pleiotropy, genome-wide association study

## Abstract

Parkinson’s disease and inflammatory bowel disease have been increasingly associated, implying shared pathophysiology. To explore biological explanations for the reported connection, we leveraged summary statistics of updated genome-wide association studies and characterized the genetic overlap between the two diseases. Aggregated genetic association data were available for 37 688 cases versus 981 372 controls for Parkinson’s disease and 25 042 cases versus 34 915 controls for inflammatory bowel disease. Genetic correlation was estimated with the high-definition likelihood method. Genetic variants with joint association to both diseases were identified by conditional false discovery rate framework and further annotated to reveal shared loci, genes, and enriched pathways. For both Crohn’s disease and ulcerative colitis, the two main subtypes of inflammatory bowel disease, we detected weak but statistically significant genetic correlations with Parkinson’s disease (Crohn’s disease: *r_g_* = 0.06, *P* = 0.01; ulcerative colitis: *r_g_* = 0.06, *P* = 0.03). A total of 1290 variants in 27 independent genomic loci were detected to associate with Parkinson’s disease and Crohn’s disease at conjunctional false discovery rate under 0.01 and 1359 variants in 15 loci were pleiotropic to Parkinson’s disease and ulcerative colitis. Among the identified pleiotropic loci, 23 are novel and have never been associated with both phenotypes. A mixture of loci conferring either same or opposing genetic effects on two phenotypes was also observed. Positional and expression quantitative trait loci mapping prioritized 296 and 253 genes for Parkinson’s disease with Crohn’s disease and ulcerative colitis, respectively, among which only <10% are differentially expressed in both colon and substantia nigra. These genes were identified to overrepresent in pathways regulating gene expression and post-translational modification beyond several immune-related pathways enriched by major histocompatibility complex genes. In conclusion, we found robust evidence for a genetic link between Parkinson’s disease and inflammatory bowel disease. The identified genetic overlap is complex at the locus and gene levels, indicating the presence of both synergistic and antagonistic pleiotropy. At the functional level, our findings implied a role of immune-centered mechanisms in the reported gut-brain connection.

## Teaser

To explore the connection between Parkinson’s disease and inflammatory bowel disease, Kang *et al*. harnessed the largest genome-wide association studies and identified 23 novel loci that have never been associated with both diseases. The discovered genetic overlap implies the presence of both synergistic and antagonistic mechanisms underlying the two diseases.

## Graphical Abstract

**Graphical abstract:**
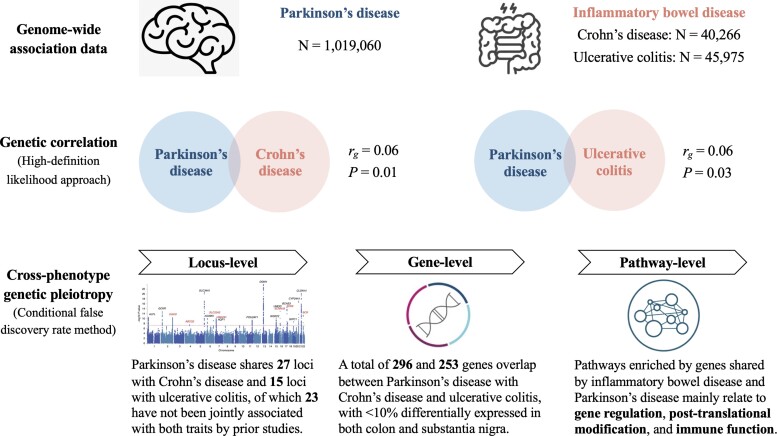

## Introduction

Parkinson’s disease is a neurodegenerative movement disorder with no curative or disease-modifying therapies, suggesting an urgent need for better understanding of disease pathophysiology to foster drug discovery. The gut–brain axis has been hypothesized to play a role in Parkinson’s disease pathogenesis, stimulating a growing body of work on the putative contribution of gastrointestinal dysfunction in Parkinson’s disease initiation.^1,2^ Inflammatory bowel disease (IBD) is a chronic intestinal inflammatory condition manifested by long-lasting diarrhoea, abdominal pain and bloody stool.^3^ Recently, a meta-analysis of nine observational studies comprising over 12 million patients demonstrated an interesting bidirectional relationship between Parkinson’s disease and IBD, where IBD as a risk factor was associated with 25–30% increase of Parkinson’s disease risk and as an outcome was 40% more likely to develop among Parkinson’s disease patients.^4^ A protective effect of anti-inflammatory medications on Parkinson’s disease among IBD patients was also reported by the authors.^4^ Together with the emerging data from subsequent epidemiological studies, these evidence converged to support a biological link between the two seemingly unrelated conditions.^5,6^

How the inflamed gut and its underlying mechanisms are intrinsically connected to Parkinson’s disease remains elusive. A sophisticated interplay between mucosal immunity and intestinal microbiota, two key drivers in IBD, has been shown to be relevant.^7–9^ Mechanistically, the inflammation-associated disruption of the intestinal epithelial barrier facilitates the translocation of microbial products from the intestinal lumen into the peripheral circulation, inducing gut dysbiosis and systemic inflammation.^10^ These events next trigger upregulation of α-synuclein (the pathogenic protein in Parkinson’s disease) expression and its abnormal aggregation in enteric neurons, as well as neuroinflammation, which promotes neurodegeneration in the brain.^11,12^ Indeed, onset of Parkinson’s disease-like neuropathology has been observed in an animal model of colitis, reinforcing the detrimental role of intestinal inflammation in neuroimmune regulation and dopaminergic neuronal function.^13^ Moreover, infection by enteric pathogens and gut dysbiosis have been both documented as common risk factors for Parkinson’s disease and IBD.^14–18^

Discovery of genetic overlap between Parkinson’s disease and IBD has shed new light into the molecular underpinning shared by the two diseases. For instance, polymorphisms of the leucine-rich repeat kinase 2 (*LRRK2*) gene have been robustly associated with susceptibility to both Parkinson’s disease and Crohn’s disease, a subtype of IBD, corroborating the crucial role of the immune system in the two conditions.^19,20^ Owing to the methodological advancement in cross-phenotype pleiotropic analyses, additional common genetic determinants of Parkinson’s disease and IBD have been identified.^21^ Hence, to further explore the Parkinson’s disease-IBD connection genetically and to systematically uncover novel mechanistic explanations for this relationship, we leveraged summary statistics from updated genome-wide association studies (GWAS) of largest sample size to date for Parkinson’s disease and IBD and characterized the genome-wide pleiotropy between the two diseases at multiple biological levels.

## Materials and methods

### Source data

Summary statistics from two GWASes was analysed in the present work. Genetic associations with Parkinson’s disease were derived from a meta-analysis of 16 case–control samples from the International Parkinson’s Disease Genomics Consortium (IPDGC) and 23andMe, Inc, comprising a total of 37 688 patients with clinically ascertained (65%) or self-reported (35%) Parkinson’s disease and 981 372 controls of European ancestry. More details about sample characteristics and the study protocol are described elsewhere.^22^

The IBD GWAS summary statistics was based on a total of 59 957 participants, combining a UK sample of 25 305 European individuals (*N*_case/control_ = 12 160/13 145) and the International Inflammatory Bowel Disease Genetics Consortium sample (*N*_case/control_ = 12 882/21 770).^23^ All IBD patients were clinically ascertained, and subtype information on Crohn’s disease or ulcerative colitis (UC) was available. Details about study participants and protocol can be found in the original publication.^23^ Given the pathophysiological and genetic differences between IBD subtypes,^24–26^ we accessed the genetic associations with both Crohn’s disease (combined *N*_case/control_ = 12 194/28 072) and UC (combined *N*_case/control_ = 12 366/33 609) and analysed the two subtypes separately throughout the study. Since the study participants in the Parkinson’s disease and IBD GWAS came from independent case–control studies, there is no overlap between the Parkinson’s disease and IBD samples.

### Statistical analysis

#### Genetic correlation analysis

**Figure 1:**
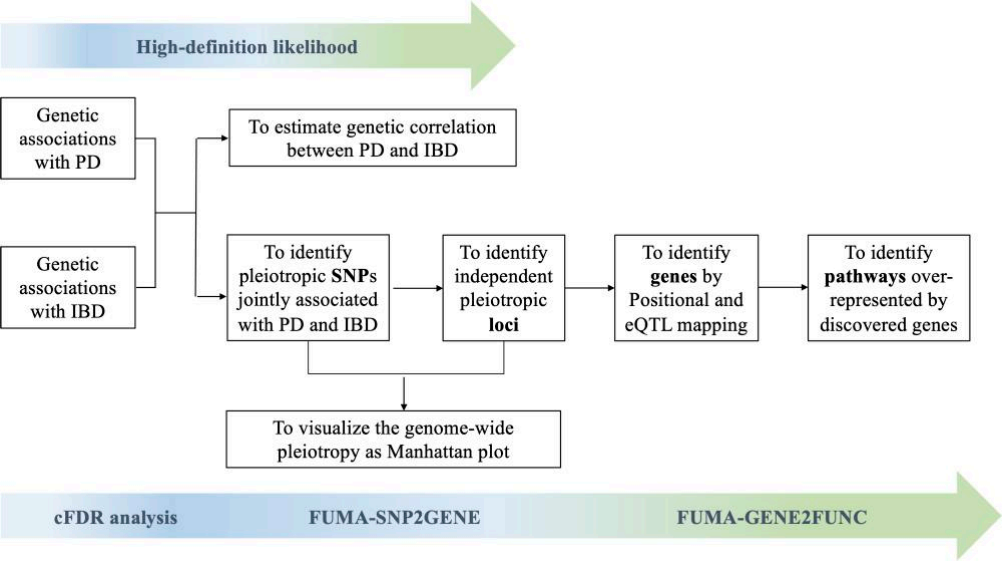
**Schematic diagram of the analytical procedures implemented in the study**.

The analytical process is displayed as a flowchart in Fig. 1. Genetic correlations (*r_g_*) between Parkinson’s disease and each IBD subtype were estimated via high-definition likelihood (HDL), a full likelihood-based extension of the conventional linkage disequilibrium (LD) score regression method that improves *r_g_* estimation precision by integrating more information on the LD structure.^27,28^ The HDL analysis was performed via the software provided by Ning *et al*.^28^ at https://github.com/zhenin/HDL/.

#### Conditional false discovery rate analysis

Genetic overlap between Parkinson’s disease and each IBD subtype was explored using the conditional false discovery rate (cFDR) framework proposed by Andreassen *et al*.^29^ First, we visualized the extent of shared genetics affecting both traits as conditional or stratified quantile–quantile (Q–Q) plots: for any single phenotype, a conventional Q–Q plot shows the quantiles of the −log_10_-transformed *P*-values from the corresponding GWAS (on the vertical axis) against the quantiles from an equally transformed uniform distribution corresponding to a global null distribution (on the horizontal axis); an upward deflection of the resulting curve from the line of identity reflects an enrichment of smaller than expected *P*-values, corresponding to a deviation from the null and the presence of a biological signal. For pairs of phenotypes, conditional Q–Q plots show multiple such quantile curves for the primary or target phenotype, where each curve corresponds to a subset of variants where the *P*-values for the secondary or conditioning phenotype are selected by increasingly stringent thresholds: in the complete absence of pleiotropy, these curves should coincide, whereas in the presence of pleiotropy, we expect to see increasing upward deflection from the global null and consequently increasing enrichment of variants associated with the primary phenotype among the subsets defined by the secondary phenotype.^29–31^

Next, we identified pleiotropic single nucleotide polymorphisms (SNPs) for Parkinson’s disease-Crohn’s disease and Parkinson’s disease-UC, respectively, by calculating the conjunctional false discovery rate (conjFDR) value for each SNP included in both Parkinson’s disease and IBD datasets. The conjFDR is based on the cFDR and can be interpreted as a conservative estimate of the false discovery rate for a given SNP being jointly associated with both phenotypes under investigation.^29^ We defined pleiotropic SNPs as those with conjFDR below a threshold of 0.01.

To assure approximate independence of the variants/*P*-values involved, we implemented LD-based random pruning for both conditional Q–Q plots and calculation of the conjFDR at *R*^2^ < 0.05 (the latter based on 100 independent iterations).^29,30^ Variants located in the major histocompatibility complex (MHC; chromosome 6: 28 477 797–33 448 354 per human genome assembly GRCh37/hg19) and microtubule-associated protein tau (MAPT; chromosome 17: 43 384 864–44 913 631 per GRCh37/hg19) regions were excluded from this step, as the known complexity of the LD structure in the two regions can make random pruning unreliable. After cFDR calculation for all SNPs in the non-MHC/MAPT regions, we then imputed the conjFDR values for variants in these two regions via *post hoc* estimation. Details about the concept and the recommended analytical protocol for cFDR analysis are reviewed by Smeland *et al*.^30^ The cFDR analysis was performed with R version 4.0.3 using the cfdr.pleio package available at https://github.com/alexploner/cfdr.pleio.

#### Functional mapping and annotation

Characterization and functional annotation of the pleiotropic SNPs identified from cFDR were performed via the FUMA web-based platform under the default setting if not otherwise specified.^32^ Using the FUMA-SNP2GENE function, we first annotated all pleiotropic SNPs via the built-in ANNOVAR tool and identified their corresponding genomic loci based on the LD pre-computed from the 1000 genome reference panel.^33,34^ Of note, we merged any pleiotropic loci identified from the MHC region into one locus and named it as ‘MHC’. The direction of genetic effects of a pleiotropic locus on Parkinson’s disease and IBD subtype was determined based on the proportion of concordant pleiotropic SNPs, defined as the variants with positive product of two association coefficients (i.e. *β*_Parkinson’s disease_ × *β*_IBD_ > 0), among the total pleiotropic SNPs within the corresponding locus. We regarded a locus containing ≤10%, 10–90%, and ≥90% of concordant SNPs as being of antagonistic, ambiguous, and concordant pleiotropy, respectively. The distribution of jointly associated SNPs and loci in different directions of pleiotropy was then visualized as Manhattan plots. To identify novel pleiotropic loci that have not been previously associated with Parkinson’s disease-Crohn’s disease or Parkinson’s disease-UC, we searched the Parkinson’s disease GWAS Locus Browser (https://pdgenetics.shinyapps.io/GWASBrowser/)^35^ and the GWAS Catalog (https://www.ebi.ac.uk/gwas/) for all shared loci informed by our cFDR analysis, considering evidence from both GWAS and cross-phenotype analysis.

Next, we proceeded with gene prioritization via positional and expression quantitative trait loci (eQTL) mapping. We selected the GTEx v8 database for eQTL mapping and restricted the tissue types to colon (including sigmoid and transverse) and substantia nigra, which are most relevant to IBD and Parkinson’s disease pathophysiology.^36^ All pleiotropic SNPs were gene-mapped using the FUMA-SNP2GENE function without any filtering per functional annotations (i.e. functional consequence or Combined Annotation Dependent Depletion, CADD score). The genes prioritized via positional and eQTL mapping were then taken forward to the FUMA-GENE2FUNC function to infer putative biological pathways by overrepresentation analysis. Since the complex gene structure in the MHC region may confound the downstream gene-set results, we performed two separate pathway analyses: one using only non-MHC genes as input and the other including both non-MHC and MHC genes. Significantly enriched gene sets or pathways were determined per FUMA-GENE2FUNC default parameters adjusting for multiple testing.

### Ethics statement

For each GWAS study included in the present work, written informed consent was received from all participants, and ethical approval was obtained from relevant ethical review boards. No additional ethical approval was required for our study because the analyses were based on summary statistics—i.e. without accessing individual-level genetic data.

## Results

**Figure 2:**
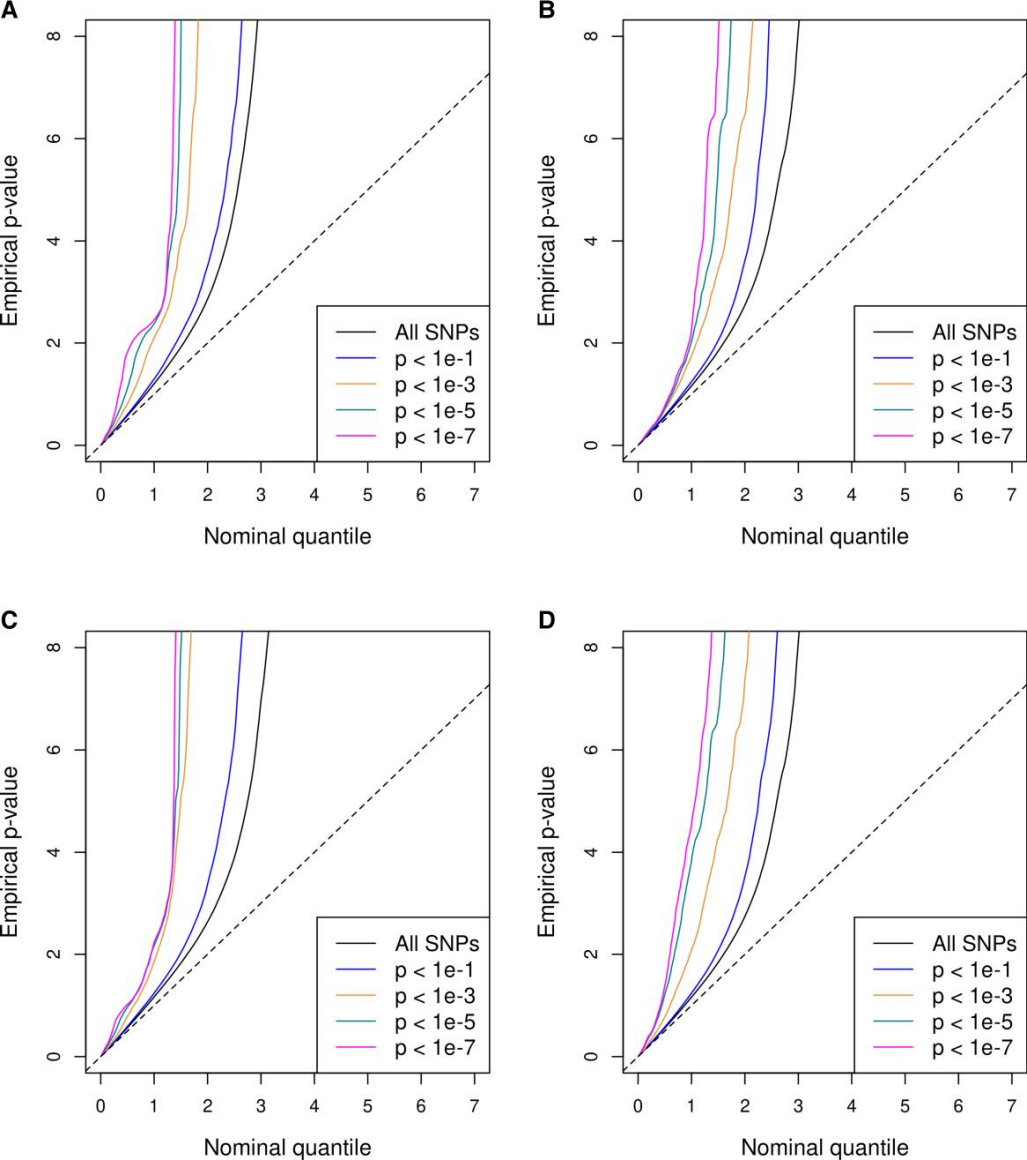
**Conditional Q–Q plots for pleiotropic enrichment between Parkinson’s disease and each subtype of IBD.** The *y*-axis denotes the −log_10_-transformed *P*-values from the GWAS of the primary trait, and the *x*-axis denotes the equally transformed uniform distribution corresponding to a global null distribution, for SNPs stratified by their *P*-values from the GWAS of the conditional trait. Pleiotropic enrichments are displayed separately for Crohn’s disease conditional on Parkinson’s disease (**A**), Parkinson’s disease conditional on Crohn’s disease (**B**), UC conditional on Parkinson’s disease (**C**), and Parkinson’s disease conditional on UC (**D**).

Weak but statistically significant genetic correlations of Parkinson’s disease with both Crohn’s disease (*r_g_* = 0.06, s.e. = 0.02; *P* = 0.01) and UC (*r_g_* = 0.06, s.e. = 0.03; *P* = 0.03) were detected by HDL (Table 1). In accordance, the successive leftward shift of curves seen in the conditional QQ plots for both trait pairs in both directions corroborated the presence of genetic overlap between Parkinson’s disease and each IBD subtype (Fig. 2). These deflections can be understood as increases in the excess of non-null SNPs for the primary phenotype when sequentially selecting sets of SNPs with stronger evidence of conditional associations. Interestingly, we noticed that the curves separated more prominently when more relaxed *P* levels were conditioned on. Once the conditional *P* cut-off reached 1 × 10^−5^ (represented by the green curves in Fig. 2), there was not much gain in association enrichment by requiring stronger evidence on conditional associations. Such trends might imply that the pleiotropy underlying Parkinson’s disease and IBD is mainly attributable to SNPs with mild to moderate levels of evidence on phenotype associations but not to those with top signals for each trait.

**Table 1:** Genetic correlations between Parkinson’s disease and each subtype of IBD

Phenotypes	*r_g_*	Standard error	*P*
Parkinson’s disease and Crohn’s disease	0.06	0.02	0.01
Parkinson’s disease and ulcerative colitis	0.06	0.03	0.03

**Figure 3:**
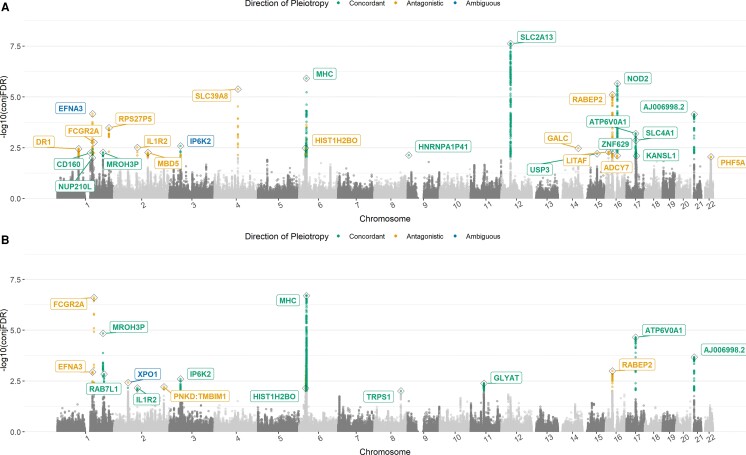
**Manhattan plots for variants and loci jointly associated with Parkinson’s disease and each subtype of IBD.** The *y*-axis denotes the −log_10_-transformed conjFDR values for each SNP. The jointly associated SNPs with conjFDR < 0.01 for Parkinson’s disease-Crohn’s disease (**A**) and Parkinson’s disease-UC (**B**) were highlighted by different colors based on their corresponding direction of pleiotropy. The jointly associated loci were labelled with the corresponding gene name, and also coloured per pleiotropic direction.

Using cFDR analysis, we identified 1290 and 1359 SNPs at conjFDR below 0.01 for Parkinson’s disease-Crohn’s disease and Parkinson’s disease-UC, respectively (Table 2). Overall, these jointly associated SNPs were mostly concordant with same genetic effects on the two phenotypes (73.6% for Parkinson’s disease-Crohn’s disease and 87.1% Parkinson’s disease-UC), located in non-exonic regions (96.8% for Parkinson’s disease-Crohn’s disease and 98.2% for Parkinson’s disease-UC), and less likely to be deleterious due to a CADD score under 12.37 (96.4% for Parkinson’s disease-Crohn’s disease and 96.0% for Parkinson’s disease-UC).^37^ After combining all pleiotropic loci in the MHC region into one locus, we mapped the jointly associated SNPs to 27 independent loci for Parkinson’s disease-Crohn’s disease and 15 for Parkinson’s disease-UC, with 10 pairwise overlapping loci (Table 3; Supplementary Tables 1 and 2). Among the 32 distinct loci in total, 23 had never been previously reported to affect both Parkinson’s disease and IBD or either of its subtypes and were therefore considered as novel shared loci (Supplementary Table 7). As displayed in the Manhattan plots, the locus-level pleiotropic patterns for Parkinson’s disease-Crohn’s disease and Parkinson’s disease-UC exhibited similarities in that the jointly associated loci were distributed widely across the entire genome and comprised a mixture of pleiotropic directions (Fig. 3). The two trait pairs however differed in their strongest association, which was in the *SLC2A13* locus on chromosome 12 (shared with *LRRK2*) for Parkinson’s disease-Crohn’s disease but in the ‘MHC’ locus on chromosome 6 for Parkinson’s disease-UC. Interestingly, 4 of the 10 pairwise common loci had different pleiotropic directions: *IL1R2* on chromosome 2 and *HIST1H2BO* on chromosome 6 were antagonistic for Parkinson’s disease-Crohn’s disease but concordant for Parkinson’s disease-UC, while *EFNA3* on chromosome 1 and *IP6K2* on chromosome 3 were ambiguous for Parkinson’s disease-Crohn’s disease but affected Parkinson’s disease-UC in opposite and same directions, respectively. Although chance finding cannot be excluded, particularly for *HIST1H2BO* where only one pleiotropic variant in total was detected for Parkinson’s disease-UC, the discrepancy of pleiotropic direction for common loci mirrors the earlier finding that Crohn’s disease and UC may be genetically distinctive.^25^

**Table 2:** Genome-wide pleiotropy for Parkinson’s disease and IBD

	Parkinson’s disease and Crohn’s disease	Parkinson’s disease and ulcerative colitis
Pleiotropic SNPs at conjFDR < 0.01		
Total	1290 (100%)	1359 (100%)
Concordant SNPs	949 (73.6%)	1184 (87.1%)
Exonic SNPs	41 (3.2%)	24 (1.8%)
Deleterious SNPs^a^	47 (3.6%)	54 (4.0%)
Independent loci identified from the pleiotropic SNPs, by direction of pleiotropy^b^		
Total	27 (100%)	15(100%)
Novel^c^	18 (66.7%)	10 (66.7%)
Concordant	13 (48.1%)	10 (66.7%)
Antagonistic	12 (44.4%)	4 (26.7%)
Ambiguous	2 (7.4%)	1 (6.7%)
Prioritized genes, by mapping method		
Total	296	253
eQTL, colon only^d^	110	88
eQTL, substantia nigra only	2	1
eQTL, colon and substantia nigra	21	21
Overrepresented gene sets with adjusted *P* < 0.05		
Including only non-MHC genes^e^	23	1
Including both non-MHC and MHC genes	72	82

a Defined as those with CADD score > 12.37.

b All pleiotropic loci in the MHC region were combined into one locus. Direction of pleiotropy for a locus is defined by the proportion of concordant SNPs divided by the total number of SNPs within the locus: ‘concordant’ if ≥90%, ‘antagonistic’ if ≤10%, and ‘ambiguous’ if 10–90%.

c Based on literature search.

d Includes sigmoid and transverse.

e Included 212 genes for Parkinson’s disease-Crohn’s disease and 136 genes for Parkinson’s disease-UC.

**Table 3:** Independent pleiotropic loci with conjFDR below 0.01

CHR	Start	End	Gene	SNP^a^	conjFDR^a^	Direction^a,b^	Ratio^a,c^
Pleiotropic loci shared by both Parkinson’s disease Parkinson’s disease-Crohn’s disease and Parkinson’s disease-UC (*N* = 10)							
1	155 033 317	155 816 085	*EFNA3*	rs35902694 rs7535292	6.70E−05 1.14E−03	Ambiguous Antagonist	4/37 0/12
1	161 075 260	161 479 745	*FCGR2A*	rs10800309 rs6658353	1.71E−03 2.49E−07	Antagonistic Antagonistic	0/2 0/17
1	200 874 375	201 032 273	*MROH3P*	rs3738255 rs12045164	5.34E−03 1.43E−05	Concordant Concordant	7/7 20/20
2	102 573 815	102 681 675	*IL1R2*	rs3218921 rs7567514	3.08E−03 6.93E−03	Antagonistic Concordant	0/3 9/9
3	48 446 237	49 749 976	*IP6K2*	rs73082337 rs9848268	2.59E−03 2.52E−03	Ambiguous Concordant	5/9 27/28
6	27 607 111	28 172 704	*HIST1H2BO*	rs200966 rs17767294	3.30E−03 7.26E−03	Antagonistic Concordant	0/12 1/1
6	29 604 683	32 961 766	*MHC*	rs1738434 rs9268556	1.22E−06 2.00E−07	Concordant Concordant	199/208 891/951
16	28 338 039	29 001 460	*RABEP2*	rs9708958 rs62036658	7.70E−06 1.01E−03	Antagonistic Antagonistic	0/148 0/81
17	40 568 094	40 824 834	*ATP6V0A1*	rs12936169 rs9897702	6.30E−04 2.22E−05	Concordant Concordant	67/67 35/35
21	16 804 330	16 841 303	*AJ006998.2*	rs1736023 rs1736023	7.11E−05 2.13E−04	Concordant Concordant	52/52 52/52
Pleiotropic loci shared by Parkinson’s disease-Crohn’s disease only (*N* = 17)							
1	93 538 949	93 895 483	*DR1*	rs11576494	3.54E−03	Antagonistic	0/66
1	145 562 881	145 718 124	*CD160*	rs744877	5.63E−03	Concordant	1/1
1	153 768 179	154 270 018	*NUP210L*	rs34305899	9.94E−03	Concordant	1/1
1	226 954 879	226 965 334	*RPS27P5*	rs10916037	3.32E−04	Antagonistic	0/14
2	148 534 374	148 956 584	*MBD5*	rs13001158	5.44E−03	Antagonistic	0/25
4	103 001 649	103 387 161	*SLC39A8*	rs13107325	4.13E−06	Antagonistic	0/14
9	4 946 261	4 950 144	*HNRNPA1P41*	rs62541503	7.30E−03	Concordant	1/1
12	39 306 511	41 036 198	*SLC2A13*	rs28370649	2.34E−08	Concordant	495/495
14	88 365 902	88 389 540	*GALC*	rs59884956	3.35E−03	Antagonistic	0/1
15	63 792 023	63 884 462	*USP3*	rs55848327	6.23E−03	Concordant	1/1
16	11 697 890	11 727 983	*LITAF*	rs72781032	5.01E−03	Antagonistic	0/5
16	30 592 110	30 877 636	*ZNF629*	rs35695082	4.53E−03	Concordant	1/1
16	50 317 816	50 329 374	*ADCY7*	rs3760013	7.71E−03	Antagonistic	0/3
16	50 715 185	50 845 533	*NOD2*	rs2067085	2.19E−06	Concordant	83/83
17	42 292 020	42 330 171	*SLC4A1*	rs7209801	1.37E−03	Concordant	30/30
17	43 463 493	44 865 603	*KANSL1*	rs62060837	7.90E−03	Concordant	2/2
22	41 637 119	42 043 585	*PHF5A*	rs9607805	8.86E−03	Antagonistic	0/2
Pleiotropic loci shared by Parkinson’s disease-UC only (*N* = 5)							
1	205 663 993	205 757 824	*RAB7L1*	rs708723	1.45E−03	Concordant	6/6
2	61 107 886	61 837 947	*XPO1*	rs9309337	3.79E−03	Ambiguous	1/2
2	219 052 546	219 191 569	*PNKD:TMBIM1*	rs2271543	6.14E−03	Antagonistic	0/3
8	116 409 058	116 437 154	*TRPS1*	rs800907	9.79E−03	Concordant	1/1
11	58 174 653	58 434 545	*GLYAT*	rs1938598	4.10E−03	Concordant	141/141

a For pleiotropic loci shared by both Parkinson’s disease-Crohn’s disease and Parkinson’s disease-UC, results are reported for Parkinson’s disease-Crohn’s disease in the upper row and for Parkinson’s disease-UC in the lower row.

b Denotes the pleiotropic direction at locus level.

c Denotes the number of pleiotropic SNPs affecting the two phenotypes in the same direction divided by the number of total pleiotropic SNPs identified from the corresponding locus.

Positional and eQTL mapping prioritized 296 and 253 genes for Parkinson’s disease-Crohn’s disease and Parkinson’s disease-UC, respectively, among which 167 pairwise overlapped (Table 2, Supplementary Tables 3 and 4). For both trait pairs, around 80% of the eQTL mapped genes are differentially expressed in colon but not in substantia nigra. Following the FUMA-GENE2FUNC procedure, 23 curated gene sets for Parkinson’s disease-Crohn’s disease and 1 for Parkinson’s disease-UC (Supplementary Table 5) were overrepresented by genes prioritized from non-MHC regions. In general, these non-MHC enriched gene sets are functionally related to gene regulation and post-translational modification. When MHC genes were re-introduced in the pathway analysis, we found 72 and 82 curated gene sets for Parkinson’s disease-Crohn’s disease and Parkinson’s disease-UC, respectively (Supplementary Table 6). These contained 11 KEGG pathways for Parkinson’s disease-Crohn’s disease, which are all related to host immunity or autoimmune diseases and are also significantly enriched by Parkinson’s disease-UC genes (Supplementary Fig. 1). Another three KEGG pathways unique to Parkinson’s disease-UC—‘natural killer cell mediated cytotoxicity’, ‘hematopoietic cell lineage’ and ‘endocytosis’—were also predominated by HLA genes in the MHC region (Supplementary Fig. 1C).

## Discussion

Despite a modest genetic correlation, we discovered robust evidence for a genetic link between Parkinson’s disease and each IBD subtype, underpinned by many shared genomic regions including 23 novel loci. The identified genetic overlap is complex at the locus and gene levels, indicating the presence of both common aetiology and antagonistic pleiotropy between Parkinson’s disease and IBD. Nonetheless, at the functional level, the Parkinson’s disease-IBD genetic overlap is featured by a predominance of gene sets regulating gene expression and post-translational modification beyond a group of immune-related pathways enriched by MHC genes.

The biological connection between Parkinson’s disease and IBD has been intensely studied.^4,19,21^ Leveraging human genetic data from the largest GWAS to date, we only detected a weak (though statistically significant) genetic correlation between Parkinson’s disease and each IBD subtype, in contrast to the pleiotropic enrichment seen in the conditional Q–Q plots. Based on our characterization of the pleiotropic direction at variant and locus levels, we suggest that this unexpectedly weak genetic correlation may be attributable to the mixture of concordant and antagonistic pleiotropy observed, which will bias the slope of the cross-trait LD score regression towards the null.^38^

At locus-level, we discovered 23 novel loci that had not been previously associated with both Parkinson’s disease and IBD, 17 of which were also novel for Parkinson’s disease. Compared with an earlier cFDR study of the pleiotropy between Parkinson’s disease and several autoimmune diseases, we replicated four loci (*MROH3P*, *HLA-DQB1*, *LRRK2*, and *MAPT*) for Parkinson’s disease-Crohn’s disease and two MHC loci for Parkinson’s disease-UC; in contrast, the previously reported loci *CCNY*, *RSPH6A* and *SYMPK* for Parkinson’s disease-Crohn’s disease and *GUCY1A3* for Parkinson’s disease-UC were not captured by our data, even when relaxing the conjFDR threshold to 0.05.^21^ We also failed to replicate *COL13A1* on chromosome 10, which had previously been indicated as pleiotropic for Parkinson’s disease and UC, based however on a Parkinson’s disease GWAS in an Amish population.^39^ Notably, among all replicated loci, *MROH3P* is an IBD risk locus that was initially nominated to be shared by Parkinson’s disease-Crohn’s disease but not Parkinson’s disease-UC.^21^ Here, we confirmed its concordant pleiotropy for Parkinson’s disease-Crohn’s disease and extended it further to Parkinson’s disease-UC. Furthermore, the association of *MROH3P* with colonic expression of *C1orf106*, an IBD susceptibility gene encoding a key protein for epithelial homeostasis, also suggests a role of intestinal barrier dysfunction in Parkinson’s disease and IBD pathogenesis.^40^ For another IBD risk locus *IL1R2*, we found conflicting pleiotropy between Parkinson’s disease-Crohn’s disease (antagonistic) and Parkinson’s disease-UC (concordant). This is contradictory to the immune regulatory role of interleukin-1 receptor 2, encoded by *IL1R2*, consistently described in both Crohn’s disease and UC.^41^ As we found no functional impact of *IL1R2* variation on gene expression in colon or substantia nigra, future research is warranted. The Parkinson’s disease risk locus at *IP6K2* is also noteworthy for its association with expression of candidate Parkinson’s disease gene *WDR6* and four other genes (*NCKIPSD*, *GMPPB*, *PRKAR2A*, and *AMT*) in both colon and substantia nigra.^42^ Intriguingly, the *IP6K2* was shown to confer ambiguous pleiotropy for Parkinson’s disease-Crohn’s disease in our present work and needs subsequent studies to follow-up.

In contrast to the complexity of locus-level pleiotropy, the gene sets shared by Parkinson’s disease and IBD are mostly related to gene regulation and post-translational modification before considering MHC genes. When MHC genes were re-introduced into analysis, the results were dominated by numerous immune-related pathways and demonstrated high concordance between Parkinson’s disease-Crohn’s disease and Parkinson’s disease-UC, in line with previous findings.^21^ However, the results from the MHC-included analysis should be interpreted with caution.

The Parkinson’s disease-IBD relationship has also been previously investigated via Mendelian randomization (MR) framework, an instrumental variable approach for causal inference. Using IBD-associated genetic variants obtained from GWAS data as instrument, neither Freuer and Meisinger^43^ nor Li and Wen^44^ found convincing evidence for a causal effect of IBD or its subtype on the risk of Parkinson’s disease. We have also been unable to replicate the putative neuroprotective effect of tumour necrosis factor inhibitors, an anti-inflammatory treatment indicated for IBD, via MR in a previous study.^45^ In contrast, a recent MR study reported preliminary evidence for causality from genetically instrumented Parkinson’s disease on IBD risk.^46^ At first glance, the lack of robust causal evidence seems to conflict the mounting data in support of observational association, genetic correlation and genetic pleiotropy between IBD and Parkinson’s disease.^4–6,13,21^ It is however important to note that neither phenotypic nor genetic association necessarily imply causation; in other words, the two diseases may co-occur, share common genetic determinants and correlate genetically without a definitive cause-and-effect relation.

Strengths of our study are the implementation of powerful statistical inference methods and the utilization of the most updated GWAS data. The reproducibility of our findings is enhanced by the choice of a conservative conjFDR threshold of 0.01. To our knowledge, we are the first to make the distinction between concordant and antagonistic pleiotropy at both variant and locus levels in research on the Parkinson’s disease-IBD connection, which enabled us to propose an explanation for the observed weak genetic correlation. The present work also has limitations. First, functional validation of detected genetic elements is beyond our scope, restricting causal interpretation of our findings. Nevertheless, we performed multi-hierarchical bioinformatic analyses to facilitate biological inference. Second, restricted by data availability, we were not able to distinguish up-regulated from down-regulated genes in the eQTL mapping. Future efforts should be made to further clarify the pleiotropic direction at higher biological hierarchies, such as gene and pathway levels. Third, as mentioned above, the strong LD in the MHC locus may bias our gene-set analysis of both non-MHC and MHC genes, restricting result comparison and interpretation.

## Conclusion

Our genetic evidence supports the notion that Parkinson’s disease and IBD are biologically connected phenotypes and indicate the immune system as a putative target for therapeutic development for both Parkinson’s disease and IBD.

## Supplementary Material

fcad002_Supplementary_Data

## Data Availability

Full summary statistics of GWAS on IBD are publicly available at GWAS Catalog: genetic associations with Crohn’s disease can be downloaded at https://www.ebi.ac.uk/gwas/studies/GCST004132 and for UC at https://www.ebi.ac.uk/gwas/studies/GCST004133. Summary statistics of Parkinson’s disease GWAS can be obtained via research project applications to 23andMe and the IPDGC. For 23andMe, the full GWAS summary statistics for the discovery data set will be made available through 23andMe to qualified researchers under an agreement with 23andMe that protects the privacy of the 23andMe participants. Please visit research.23andme.com/collaborate/for more information and to apply to access the data.

## Abbreviations

CADD =: combined annotation dependent depletion
cFDR =: conditional false discovery rate
conjFDR =: conjunctional false discovery rate
eQTL =: expression quantitative trait loci
GWAS =: genome-wide association study
HDL =: high-definition likelihood
IBD =: inflammatory bowel disease
IPDGC =: International Parkinson’s Disease Genomics Consortium
LD =: linkage disequilibrium
LRRK2 =: leucine-rich repeat kinase 2
MAPT =: microtubule-associated protein tau
MHC =: major histocompatibility complex
Q–Q =: quantile–quantile
SNP =: single nucleotide polymorphism
UC =: ulcerative colitis
