# Supplementary material for: Genetic overlap between Parkinson’s disease and inflammatory bowel disease
Source: Brain Commun. 2023 Jan 3;5(1):fcad002. doi: 10.1093/braincomms/fcad002 (PMC9847552; doi:10.1093/braincomms/fcad002)
Supplement: fcad002_Supplementary_Data [file fcad002_supplementary_data.zip › Supplementary Figure 1.pdf]

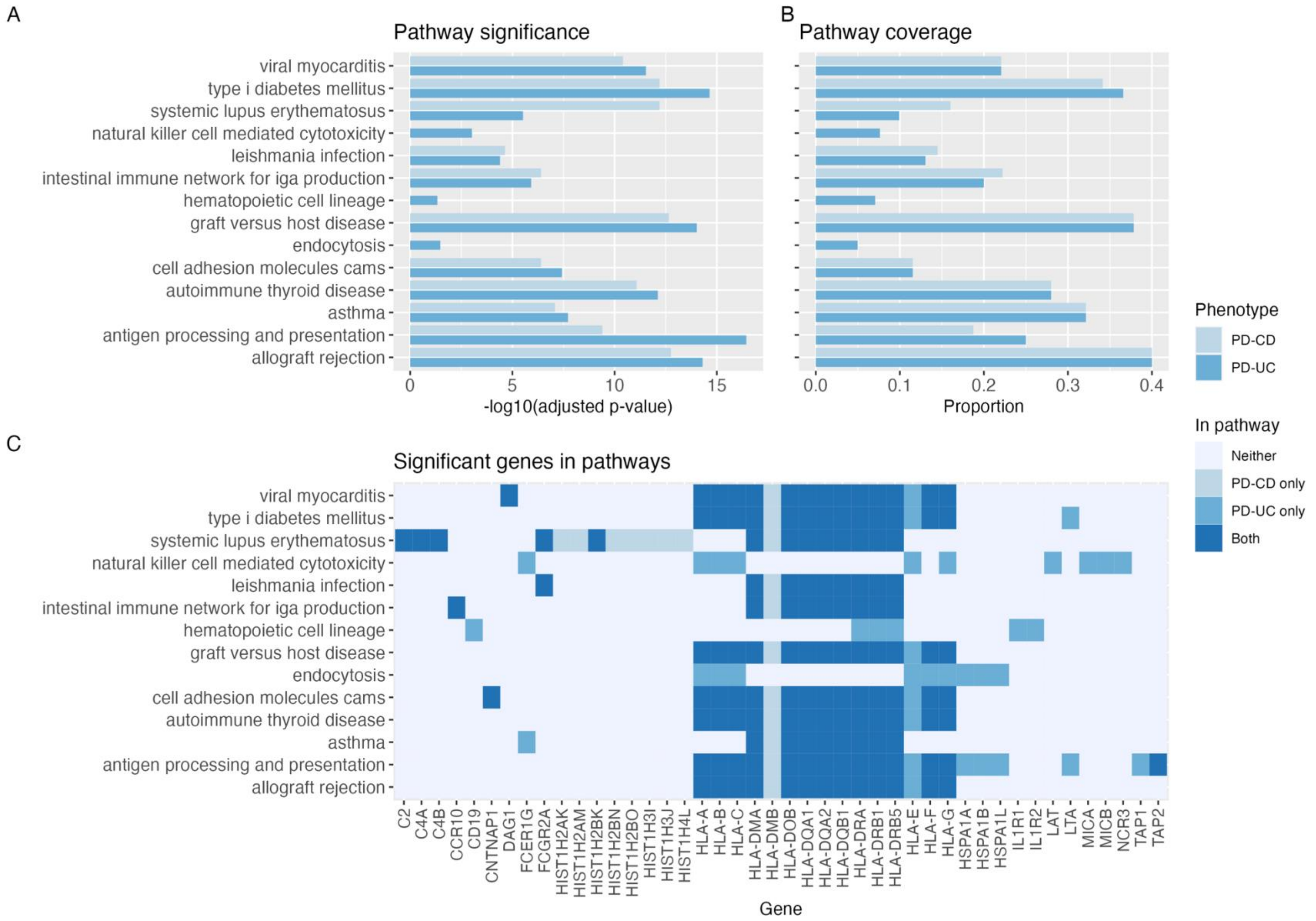
A
Pathway significance
B
Pathway coverage
viral myocarditis
type i diabetes mellitus
systemic lupus erythematosus
natural killer cell mediated cytotoxicity
leishmania infection
intestinal immune network for iga production
hematopoietic cell lineage
graft versus host disease
endocytosis
cell adhesion molecules cams
autoimmune thyroid disease
asthma
antigen processing and presentation
allograft rejection
0
5
10
15
-log10(adjusted p-value)
0.0
0.1
0.2
0.3
0.4
Proportion
Phenotype
PD-CD
PD-UC
In pathway
Neither
PD-CD only
PD-UC only
Both
C
Significant genes in pathways
C2
C4A
C4B
CCR10
CD19
CNTNAP1
DAG1
FCER1G
FCGR2A
HIST1H2AK
HIST1H2AM
HIST1H2BK
HIST1H2BN
HIST1H2BO
HIST1H3I
HIST1H3J
HIST1H4L
HLA-A
HLA-B
HLA-C
HLA-DMA
HLA-DMB
HLA-DOB
HLA-DQA1
HLA-DQA2
HLA-DQB1
HLA-DRA
HLA-DRB1
HLA-DRB5
HLA-E
HLA-F
HLA-G
HSPA1A
HSPA1B
HSPA1L
IL1R1
IL1R2
LAT
LTA
MICA
MICB
NCR3
TAP1
TAP2
Gene

Supplementary Figure 1 The KEGG pathways identified as overrepresented by FUMA-prioritized genes for Parkinson's disease and Crohn's disease or ulcerative colitis, *including* genes mapped from the MHC region.

(A) Statistical significance of identified KEGG pathways; bars represent -log10-transformed and Bonferroni-adjusted *p*-values.

(B) Coverage of identified KEGG pathways; bars represent the proportion of the prioritized genes among all genes in the pathway.

(C) Overview of prioritized genes covered by identified KEGG pathways; colors indicate statistical significance (as Bonferroni-adjusted *p*-value from the gene-set enrichment analysis < 0.05) of a gene (horizontal axis) in a corresponding KEGG pathway (vertical axis) by IBD subtype.

Abbreviations: PD, Parkinson's disease; CD, Crohn's disease; UC, ulcerative colitis.
